# Personality disorder diagnoses in UK Autistic people: Evidence from a matched cohort study

**DOI:** 10.1177/13623613251414911

**Published:** 2026-02-27

**Authors:** Elizabeth O’Nions, Jude Brown, Joshua EJ Buckman, Rebecca Charlton, Claudia Cooper, Céline El Baou, Francesca Happé, Sarah Hoare, Dan Lewer, Cathie Long, Jill Manthorpe, Douglas GJ McKechnie, Marcus Richards, Rob Saunders, Will Mandy, Joshua Stott

**Affiliations:** 1 Bradford Teaching Hospitals NHS Foundation Trust, UK; 2 National Autistic Society, UK; 3 University College London, UK; 4 iCope – Camden & Islington NHS Foundation Trust, UK; 5 Goldsmiths, University of London, UK; 6 Queen Mary, University of London, UK; 7 King’s College London, UK; 8 Independent Researcher, Cardigan, UK

**Keywords:** autism, borderline personality disorder, emotionally unstable personality disorder, personality disorder, primary care

## Abstract

Clinical accounts and cohort studies suggest that Autistic people are disproportionately likely to be diagnosed with personality disorder. We conducted a cohort study of adults diagnosed Autistic drawn from the IQVIA Medical Research Database, with follow-up from 1 January 2000 to 16 January 2019. We included a comparison group without diagnosed autism, matched (1:10) by age, sex and primary care practice. We included 22,112 Autistic adults, of whom 6437 (29.1%) had a diagnosis of intellectual disability. Median age was 20.36 (interquartile range: 18.0–28.5), and 16,881 (76.3%) were men. The rate of new personality disorder diagnosis in Autistic people without intellectual disability was 4.8 (3.5–6.7) times higher for Autistic versus comparison men, and 4.6 (3.1–6.8) times higher for Autistic versus comparison women. For Autistic participants with intellectual disability, the rate was 2.0 (1.0–3.7) times higher for Autistic versus comparison men and 8.3 (4.0–17.2) times higher for Autistic versus comparison women. The estimated rate of new personality disorder diagnosis for Autistic people aged 20 increased from 14.67 (95% confidence interval: 10.4–20.8) per 10,000 person-years in 2009 to 22.43 (95% confidence interval: 13.9–36.3) in 2019. The findings indicate that personality disorder diagnoses are more common in Autistic people and increased overall in women from 2000 to 2019.

**Lay abstract:**

Several research studies have suggested that Autistic people are more likely to be diagnosed with personality disorder than people who are not Autistic. We compared rates of personality disorder diagnoses between Autistic people and a comparison group of people not diagnosed Autistic using anonymised data collected by UK primary care practitioners for participants registered at a primary care (general practitioner) practice sometime between 1 January 2000 to 16 January 2019. The comparison group of people in the community who did not have an autism diagnosis were of the same age, sex and registered at the same primary care practice as their matched Autistic participant, with 10 times as many matched participants as Autistic participants. We included 22,112 Autistic adults, of whom 6437 (29.1%) had a diagnosis of intellectual disability. Median age was 20.36 years, and most, 16,881 (76.3%), were men. We included 221,120 comparison adults. New personality disorder diagnoses were more than four times as common for Autistic men and women without an intellectual disability compared to men and women in the comparison group. For Autistic participants with an intellectual disability, the rate was twice as high for Autistic versus comparison men and 8 times higher for Autistic versus comparison women. Between 2000 and 2019, there was an increase in the rate of new personality disorder diagnoses among Autistic people, and in women. The findings highlight the need for further investigation into reasons for this increase.

## Introduction

Autism is a lifelong neurodevelopmental condition present from birth, which impacts how a person relates to others and perceives the world. Diagnostic criteria include social communication and social interaction differences, plus highly focused and repetitive patterns of behaviours, interests and activities (World Health Organization, 2019). Globally, between 1% and 3% of the population may be Autistic (Delobel-Ayoub et al., 2020; Zeidan et al., 2022). Autistic people have different levels and types of support needs, with around 1 in 10 estimated to have at least a moderate intellectual disability (ID) (T. S. Brugha et al., 2016). Population-based studies suggest that approximately three times as many men as women are Autistic (Loomes et al., 2017). Autism is more likely to be mis- and/or late-diagnosed in women (Begeer et al., 2013; Kentrou et al., 2021, 2024), gender-diverse people (McQuaid et al., 2024) and people from minoritised ethnicities (Kelly et al., 2019; Straiton & Sridhar, 2022). It is also underdiagnosed in adult and particularly older-adult populations (O’Nions, Petersen, et al., 2023; Tromans & Brugha, 2022; Tromans et al., 2018).

According to International Classification of Diseases, 11th Revision (ICD-11), personality disorders are impairments in a person’s sense of identity, self-worth, capacity for self-direction, ability to develop and maintain close and mutually satisfying relationships, understand others’ perspectives and manage conflict in relationships (World Health Organization, 2019). Other characteristics attributed to personality disorder include ‘inflexible or poorly regulated patterns of cognition, emotional experience, emotional expression and behaviour’ (World Health Organization, 2019). A global meta-analysis suggested that 7.8% of the population meet cut-offs for personality disorders based on interviews or screening questionnaires (Winsper et al., 2020), though the prevalence of diagnosed personality disorder in the United Kingdom is much lower (Treagust et al., 2022).

Difficulty diagnosing autism in adults, changes to diagnostic criteria and ongoing limitations in access to autism diagnosis for people who grew up before the recent increase in autism awareness mean that many Autistic adults are undiagnosed (O’Nions, Petersen et al., 2023; Russell et al., 2022) or misdiagnosed (Kentrou et al., 2024; T. Brugha et al., 2020). Autism characteristics may be confused with symptoms of psychiatric disorders, and particularly with personality disorders, by mental health professionals. Clinical accounts (Eaton, 2023; Iversen & Kildahl, 2022), self-report studies (Au-Yeung et al., 2019; Kentrou et al., 2021, 2024) and observational studies of routine health data (Gilmore et al., 2021; Hand, Angell, et al., 2020; Schendel et al., 2016) indicate that Autistic people are disproportionately likely to be diagnosed with a personality disorder. Features attributed to personality disorder overlap with autism characteristics: among 47 Autistic adults accessing psychiatric services in Sweden, more than a quarter were deemed to meet criteria for paranoid, schizoid, avoidant and obsessive compulsive personality disorders (Anckarsäter et al., 2006), classifications that were removed from ICD-11 due to a lack of evidence for their validity (World Health Organization, 2019). Among 54 young Swedish adults diagnosed with Asperger syndrome, 65% of males and 32% of females were deemed to meet criteria for a personality disorder (in most cases, schizoid, avoidant or obsessive compulsive personality disorder) (Lugnegård et al., 2012).

Diagnoses of emotionally unstable or borderline personality disorder (EUPD or BPD), reportedly characterised by self-harm and emotional instability (World Health Organization, 2019), may be particularly common because Autistic people are more likely than non-Autistic people to experience traumatic events that can trigger mental health crises (Griffiths et al., 2019; Stewart et al., 2022). Among 84 Autistic people in Sweden accessing psychiatry services, the median score for BPD in Autistic women (*n* = 39) was above the clinical cut-off of 6 on a screening tool (median in women: 6.5; range: 0–9.0; median in men = 4; range 0–9) (E. Rydén & Bejerot, 2008), and 13.5% had a prior diagnosis of BPD (E. Rydén & Bejerot, 2008). In a UK study of 98 adult mental health service users assessed for autism, 5 of the 11 women (45%) identified as Autistic had a pre-existing diagnosis of BPD, but none of the 8 men (T. Brugha et al., 2020). One clinic-based Swedish study that included 117 Autistic people reported that 5% of males and 15% of females met criteria for BPD diagnosis (Hofvander et al., 2009). A meta-analysis reported a pooled prevalence of 4% for diagnoses of BPD amoung community-living Autistic participants (May et al., 2021).

Multiple accounts explain the apparent overlap between autism characteristics and personality problems (e.g., Zavlis & Tyrer, 2024). Attribution of autism characteristics to an inherent personality problem is stigmatising and potentially increases the likelihood of inappropriate treatment (Eaton, 2023; Gillett et al., 2023), exclusion from services (L. Sheehan et al., 2016; Sulzer, 2015) and of not being considered deserving of care (Lewis & Appleby, 1988). The stigma associated with personality disorder may also deter people from seeking healthcare when they need it (Bartsch et al., 2016; L. Sheehan et al., 2016). Although a small number of studies report positive impacts of a BPD diagnosis, including a sense of relief and realisation, studies (reviewed in Lester et al. (2020); Ring and Lawn (2019)) also indicate that people with this diagnosis experience exclusion in healthcare settings, and are more likely to have their medical needs disregarded (Mulder & Tyrer, 2023), in part due to negative attitudes towards personality disorder (Sulzer, 2015).

To understand the extent of diagnosis of personality disorder, including EUPD/BPD, among Autistic people, we aimed to explore the prevalence of these diagnoses and the rate of incident diagnoses in Autistic people relative to a population-based comparison group. This study aimed to establish whether Autistic men and women are more likely than the general population to have received (a) any personality disorder diagnosis and (b) an EUPD or BPD diagnosis, using data from UK electronic primary care records. We conducted these analyses separately for men and women, and for Autistic people with and without ID. We also examined time trends in personality disorder diagnosis in Autistic and non-Autistic people, and in men and women between 2000 and 2019.

## Methods

### Study design

A matched retrospective cohort study.

### Setting

This study used UK electronic primary care health records from IQVIA Medical Research Data (IMRD). This incorporates data from THIN, a Cegedim Database. Reference made to THIN is intended to be descriptive of the data asset licenced by IQVIA. IMRD contains anonymised electronic health records extracted directly from primary care computer systems from 794 UK primary care practices (c. 10% of all practices) and is approximately representative of the UK population (Blak et al., 2011).

In the United Kingdom, almost all of the population are registered with an NHS primary care practice and access is free of charge (NHS Digital, n.d.). Observations and diagnoses are captured in a person’s medical records using a structured system of clinical codes applied by primary care practitioners. Non-emergency secondary and specialist care is mostly accessed via referral by a primary care general practitioner (GP) (family doctor). Diagnoses made in secondary care are communicated to the patient’s GP and recorded in their records by practice staff. Therefore, primary care records function as a repository for an individual’s health-related information. Records are analysed from the time-point where the practice met a minimum threshold for quality of electronic record-keeping (Horsfall et al., 2013; Maguire et al., 2009).

### Ethical approval

IMRD holds ethical approval to collect and supply data for research purposes from the NHS London – South East Research Ethics Committee (reference 18/LO/0441). Use of the IMRD for this study was obtained from and approved by IQVIA World Publications Scientific Review Committee in June 2021 (reference 21SRC014).

### Study population

We included two cohorts: adults diagnosed with autism but not ID, and adults diagnosed with both autism and ID. Autism diagnoses were identified from the presence of a diagnostic label indicative of an autism spectrum condition (e.g. autism, Asperger’s, pervasive developmental disorder) in the person’s medical record. The code-list was based on previously published studies (Alfageh et al., 2020; O’Nions, Lewer, et al., 2023; R. Sheehan et al., 2015). ID diagnoses were identified from the presence of a label indicative of ID (e.g. On learning disability register, Learning disability NOS) (Alfageh et al., 2020; O’Nions, Petersen, et al., 2023; R. Sheehan et al., 2015). Code-lists are provided in a Supplemental file. For Autistic participants, the cohort entry date (i.e. the start of their observation period) was the latest of the following dates: the date of their autism diagnosis (if there was a diagnosis of both autism and ID; the date of the later diagnosis); the date that the primary care practice where the participant was registered met quality criteria for electronic healthcare record-keeping (acceptable computer usage, Horsfall et al., 2013; acceptable mortality recording Maguire et al., 2009); the participant’s date of registration at the practice +6 months; the date that the participant’s primary care practice began contributing data to IMRD; and 1 January 2000. The participant’s date of cohort exit (i.e. the end of their observation period) was the earliest of their date of death (if applicable); the date of their deregistration from the practice; the date that their practice no longer contributed data to IMRD; and 16 January 2019.

For each participant diagnosed Autistic, we sampled 10 comparison participants matched by age, sex and primary care practice. We did this by randomly sampling patients from IMRD who did not have an autism or an ID diagnosis on the date of cohort entry for each Autistic participant. We then assigned each comparison participant the same cohort entry date as their corresponding Autistic participant. This approach avoids introducing immortal time bias, as sampling at cohort entry is not conditional on diagnoses recorded in the future (Ohneberg et al., 2019). Supplemental eFigures 1 and 2 describe the identification of eligible Autistic and comparison participants and cohort entry and exit dates.

### Study variables

Diagnostic codes identifying (a) any personality disorder diagnosis and (b) an EUPD or BPD diagnosis were identified by conducting a search of relevant ICD-10 terms and by cross-referencing the search results to Read codes from existing code-lists (Abel et al., 2019; Doyle et al., 2016; Kuan et al., 2019). The list incorporated now-obsolete terms, such as neurotic personality disorder, as these may still appear on people’s medical records. For EUPD or BPD, we included terms that fell within the remit of the EUPD category within ICD-10 (World Health Organization, 1990), that is, aggressive and explosive personality disorder (see Supplementary files for all code-lists). Code-lists were reviewed by clinicians and subject-matter experts within our team.

### Statistical analysis

We estimated crude incidence of new diagnoses by dividing the number of first diagnoses by the person-time-at-risk, for Autistic people with and without ID and their respective comparison groups. Individuals who had a pre-existing record of a personality disorder at the start of the observation period were excluded from the analysis of the rate of new diagnoses (so a maximum of one diagnosis for each participant was counted when estimating rates). If participants received a new diagnosis of personality disorder during the observation period, we then censored observation on that date. The numbers of individuals with a pre-existing personality disorder diagnosis at cohort entry (who were excluded from incidence calculations) are provided in Supplemental eTable 1.^1^ We used logistic regression to compare the number of participants in the Autistic versus comparison groups with a pre-existing personality disorder diagnosis. We used Poisson regression to estimate incidence rate ratios (IRRs) comparing the incidence of personality disorder for Autistic people to that for the comparison group. In these models, personality disorder diagnosis was the dependent variable, and the independent variables were the presence of an autism diagnosis, age (treated as time-varying, with linear and quadratic terms), calendar year and an offset for the log observation time.

We also estimated rates of new diagnoses of personality disorder by calendar year for Autistic and comparison groups, stratified by sex and ID status. We standardised these incidence rates to age 20 and calendar year 2009. This allowed us to adjust the incidence rates for time trends in usage of personality disorder diagnosis present in the data, which affected the crude incidences differently given that a larger proportion of Autistic people with ID joined the cohort prior to 2010 (Tables 1 and 2). We also estimated a modelled rate of new personality disorder diagnoses by calendar year for (a) Autistic and non-Autistic participants (pooled across sexes) and (b) men and women (pooled across Autistic and comparison groups) standardised to age 20.

**Table 1.** Characteristics of Autistic participants without an intellectual disability and their respective matched comparison groups.

	Men		Women	
	Autistic	Comparison group	Autistic	Comparison group
Total participants	12,041	120,410	3634	36,340
Number of primary care practices	770	770	676	676
*Age at cohort entry*				
Median age at entry (IQR)	18.84 (18.00–25.79)	18.84 (18.00–25.79)	20.70 (18.00–29.00)	20.70 (18.00–29.00)
18–24 years	8832 (73.35)	88,320 (73.35)	2423 (66.68)	24,230 (66.68)
25–34 years	1612 (13.39)	16,120 (13.39)	564 (15.52)	5640 (15.52)
35–44 years	804 (6.68)	8040 (6.68)	336 (9.25)	3360 (9.25)
45–54 years	513 (4.26)	5130 (4.26)	223 (6.14)	2230 (6.14)
55–64 years	214 (1.78)	2140 (1.78)	68 (1.87)	680 (1.87)
65+ years	66 (0.55)	660 (0.55)	20 (0.55)	200 (0.55)
Median years of observation (IQR)	2.29 (0.91–4.54)	2.54 (1.08–5.11)	1.83 (0.77–3.64)	2.07 (0.86–4.21)
*Year of cohort entry (%)*				
2000–2010	2500 (20.76)	25,000 (20.76)	574 (15.80)	5740 (15.80)
2010–2019	9541 (79.24)	95,410 (79.24)	3060 (84.20)	30,600 (84.20)
*Socioeconomic deprivation (Townsend score) (%)*				
1: least deprived	1867 (15.51)	21,702 (18.02)	540 (14.86)	6355 (17.49)
2	1707 (14.18)	19,996 (16.61)	558 (15.35)	5795 (15.95)
3	2119 (17.60)	21,885 (18.18)	607 (16.70)	6544 (18.01)
4	2057 (17.08)	19,836 (16.47)	592 (16.29)	5958 (16.40)
5: most deprived	1505 (12.50)	13,763 (11.43)	439 (12.08)	4209 (11.58)
Missing	2786 (23.14)	23,228 (19.29)	898 (24.71)	7479 (20.58)

**Table 2.** Characteristics of Autistic participants with an intellectual disability and their respective matched comparison groups.

	Men		Women	
	Autistic	Comparison group	Autistic	Comparison group
Total participants	4840	48,400	1597	15,970
Number of primary care practices	714	714	548	548
*Age at cohort entry*				
Median age at entry (IQR)	22.51 (18.58–33.13)	22.51 (18.58–33.13)	24.50 (19.50–36.08)	24.50 (19.50–36.08)
18–24 years	2900 (59.92)	29,000 (59.92)	820 (51.35)	8200 (51.35)
25–34 years	834 (17.23)	8340 (17.23)	353 (22.10)	3530 (22.10)
35–44 years	537 (11.10)	5370 (11.10)	205 (12.84)	2050 (12.84)
45–54 years	360 (7.44)	3600 (7.44)	144 (9.02)	1440 (9.02)
55–64 years	154 (3.18)	1540 (3.18)	50 (3.13)	500 (3.13)
65+ years	55 (1.14)	550 (1.14)	25 (1.57)	250 (1.57)
Median length of observation period (IQR)	3.10 (1.29–6.18)	3.45 (1.43–6.36)	2.94 (1.17–6.38)	3.03 (1.19–6.34)
*Year of cohort entry*				
2000–2010	1578 (32.60)	15,780 (32.60)	548 (34.31)	5480 (34.31)
2010–2019	3262 (67.40)	32,620 (67.40)	1049 (65.69)	10,490 (65.69)
*Socioeconomic deprivation (Townsend score) (%)*				
1: least deprived	736 (15.21)	8986 (18.57)	241 (15.09)	3078 (19.27)
2	804 (16.61)	8561 (17.69)	269 (16.84)	2697 (16.89)
3	979 (20.23)	9014 (18.62)	331 (20.73)	2866 (17.95)
4	775 (16.01)	8127 (16.79)	237 (14.84)	2711 (16.98)
5: most deprived	563 (11.63)	5563 (11.49)	197 (12.34)	2075 (12.99)
Missing	983 (20.31)	8149 (16.84)	322 (20.16)	2543 (15.92)

Data preparation and analysis were performed using Stata 16 (StataCorp, 2019) and R version 4.3.2 (R Core Team, 2023).

### Patient and public involvement

Autistic adults and clinicians working in services supporting them were involved in the design and conduct of this research. Four Autistic adults provided consultancy via an Experts by Experience Steering Group, facilitated by The National Autistic Society. Their feedback informed the preparation of this manuscript.

### Role of the funding source

The funders of the study had no role in study design, data collection, data analysis, data interpretation or writing of the paper.

## Results

### Participants

We identified 15,675 adults with an autism diagnosis who did not have diagnosed ID prior to cohort exit; 6437 adults diagnosed Autistic with concurrent diagnosed ID, and 221,120 matched comparison participants.^2^

Most Autistic adults in the cohort were men (12,041 of those without ID (76.8%) and 4840 of those with ID (75.2%)).^3^ The majority were aged between 18 and 24 years old at cohort entry; 11,255 of those without ID (71.8%) and 3720 of those with ID (57.8%). Most Autistic adults without ID entered the cohort between 2010 and 2019 (12,601; 80.4%), compared to 4311 (67.0%) of Autistic people with ID, likely reflecting the increased rate of autism diagnoses in people without ID in recent years (O’Nions, Petersen, et al., 2023). Median age at cohort entry for Autistic adults was 18.8 years (interquartile range (IQR): 18.0–25.8) for Autistic men without ID, and 20.7 years (IQR: 18.0–29.0) for Autistic women without ID. Median age at cohort entry was 22.5 years (IQR: 18.6–33.1) for Autistic men with ID, and 24.5 years (IQR: 19.5–36.1) for Autistic women with ID, likely reflecting delayed identification of autism in women. Further demographic information is provided in Tables 1 and 2.

### Prevalent personality disorder diagnoses

Autistic men without ID were 14.0 (95% confidence interval (CI): 11.8–16.5) times more likely to have a pre-existing personality disorder diagnosis, and 14.6 (95% CI: 9.8–21.8) times more likely to have a pre-existing EUPD or BPD diagnosis relative to the comparison group at cohort entry (Supplemental eTable 1). The proportion of Autistic men without ID with a pre-existing personality disorder diagnosis was 2.7% (vs 0.2% in the comparison group), and with an EUPD or BPD diagnosis was 0.5% (vs 0.03% in the comparison group). Autistic women without ID were 14.9 (95% CI: 12.2–18.3) times as likely to have a pre-existing personality disorder diagnosis, and 12.9 (95% CI: 9.6–17.3) times as likely to have an EUPD or BPD diagnosis. The proportion of Autistic women without ID with a pre-existing personality disorder diagnosis was 6.1% (vs 0.4% in the comparison group), and with an EUPD or BPD diagnosis was 2.8% (vs 0.2% in the comparison group).

Autistic men with ID were 7.4 (95% CI: 5.8–9.3) times as likely to have a pre-existing personality disorder diagnosis, and 8.0 (95% CI: 4.5–14.4) times as likely to have a pre-existing EUPD or BPD diagnosis relative to the comparison group (Supplemental eTable 1). The proportion of Autistic men with ID with a pre-existing personality disorder diagnosis was 2.5% (vs 0.3% in the comparison group), and with an EUPD or BPD diagnosis was 0.4% (vs 0.05% in the comparison group). Autistic women with ID were 6.5 (95% CI: 4.6–9.2) times as likely to have a pre-existing personality disorder diagnosis, and 5.2 (95% CI: 2.6–10.5) times as likely to have an EUPD or BPD diagnosis. The proportion of Autistic women with ID with a pre-existing personality disorder diagnosis was 3.1% (vs 0.5% in the comparison group), and with an EUPD or BPD diagnosis was 0.8% (vs 0.1% in the comparison group).

### Incident personality disorder diagnoses

Crude incidence rates of new diagnoses of personality disorders, plus modelled rates with age fixed at 20 and year fixed at 2009, are presented in Table 3. IRRs comparing Autistic adults with and without ID to their respective comparison groups are also shown in Table 3.

**Table 3.** Rates of new personality disorder diagnoses for Autistic people with and without intellectual disability, and their respective comparison groups.

Intellectual disability	Sex	PD type	Autistic participants			Comparison group without autism or intellectual disability			Adjusted rate ratio^b^ (95% CI)
			Incident PD diagnoses/participants	Crude rate per 10,000 PYAR (95% CI)	Standardised rate per 10,000 PYAR^a^ (95% CI)	Incident PD diagnoses/participants	Crude rate per 10,000 PYAR (95% CI)	Standardised rate per 10,000 PYAR^a^ (95% CI)	
No	Men	Any	53/11,715	14.07 (10.54–18.40)	11.65 (8.82–15.39)	123/120,176	2.89 (2.40–3.44)	2.46 (1.95–3.09)	4.83 (3.50–6.67)
		BPD	13/11,981	3.34 (1.78–5.71)	1.98 (1.16–3.38)	42/120,369	0.98 (0.71–1.33)	0.46 (0.29–0.74)	3.35 (1.80–6.24)
	Women	Any	35/3413	36.82 (25.65–51.21)	26.14 (19.27–35.46)	84/36,192	7.57 (6.04–9.38)	5.51 (4.25–7.15)	4.58 (3.09–6.80)
		BPD	25/3532	25.22 (16.32–37.24)	8.42 (4.90–14.45)	53/36,261	4.77 (3.57–6.23)	1.95 (1.21–3.16)	5.04 (3.13–8.12)
Yes	Men	Any	11/4720	5.51 (2.75–9.85)	6.42 (4.12–9.98)	59/48,237	2.80 (2.13–3.61)	1.95 (1.45–2.61)	1.95 (1.03–3.72)
		BPD	2/4820	0.98 (0.12–3.53)	1.44 (0.71–2.92)	26/48,375	1.23 (0.80–1.80)	0.38 (0.22–0.65)	0.80 (0.19–3.36)
	Women	Any	13/1547	19.49 (10.38–33.32)	14.40 (9.13–22.70)	16/15,893	2.33 (1.33–3.79)	4.37 (3.19–5.98)	8.26 (3.97–17.17)
		BPD	10/1585	14.66 (7.03–26.96)	6.13 (3.02–12.42)	6/15,947	0.87 (0.32–1.90)	1.61 (0.93–2.78)	16.58 (6.02–45.62)

PD: personality disorder; BPD: borderline personality disorder; PYAR: person-years at risk; CI: confidence interval.

a Standardised to age 20 and calendar year 2009.

b Adjusted for age, age^2^ and calendar year.

Autistic participants had higher incidences of new personality disorder diagnoses (Table 3). Standardised incidences indicate a higher likelihood of personality disorder diagnoses among Autistic women compared to Autistic men and a similar pattern in comparison women versus comparison men. For Autistic people without ID, the relative likelihood of being diagnosed with personality disorder compared to the comparison group was similarly elevated for Autistic men and women. During follow-up, Autistic men without ID were 4.8 times as likely to get a new personality disorder diagnosis versus men in the comparison group (95% CI: 3.5–6.7), and 3.4 times as likely to get a new EUPD or BPD diagnosis (95% CI: 3.4 (1.8–6.2)). Autistic women without ID were 4.6 times as likely to get a new personality disorder diagnosis versus women in the comparison group (95% CI: 3.1–6.8), and 5.0 times as likely to get a new diagnosis of EUPD or BPD (95% CI: 3.1–8.1)).

Among Autistic people with ID, women were disproportionately likely to be diagnosed with a personality disorder compared to men. Autistic men with ID were 2.0 times as likely to get a new personality disorder diagnosis versus comparison men (95% CI: 1.0–3.7) but were not significantly more or less likely to get an EUPD or BPD diagnosis (IRR: 0.8 (95% CI: 0.2–3.4)). Autistic women with ID were 8.3 times as likely to get a new personality disorder diagnosis relative to comparison women (95% CI: 4.0–17.2), and 16.6 times as likely to get a new EUPD or BPD diagnosis (95% CI: 6.0–45.6).

### Time trends

**Figure 1.**
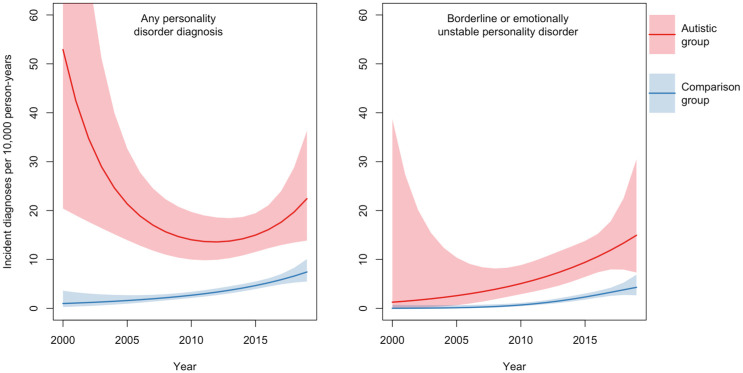
Time trends in the incidence of personality disorder diagnoses, standardised to age 20, from 2000 to 2019, with 95% confidence intervals stratified by group (Autistic vs comparison participants).

We used Poisson regression to estimate standardised incidence rates by calendar year with age fixed at 20 for Autistic and comparison participants separately, pooling data from men and women (Figure 1). The rate of new personality disorder diagnoses among Autistic participants decreased from 52.9 (95% CI: 20.4–137.1) per 10,000 person-years in 2000 to 14.7 (10.4–20.8) in 2009, then increased to 22.4 (13.9–36.3) in 2019 (Supplemental eTable 1). Possible reasons for this ‘U’ shape are discussed below. The incidence of EUPD or BPD personality disorder diagnoses among Autistic participants increased from 2000 to 2019, although again the trend is imprecise in earlier years. Incidence of all personality disorder diagnoses increased in the non-Autistic comparison groups, albeit at lower absolute rates than in the Autistic groups.

**Figure 2.**
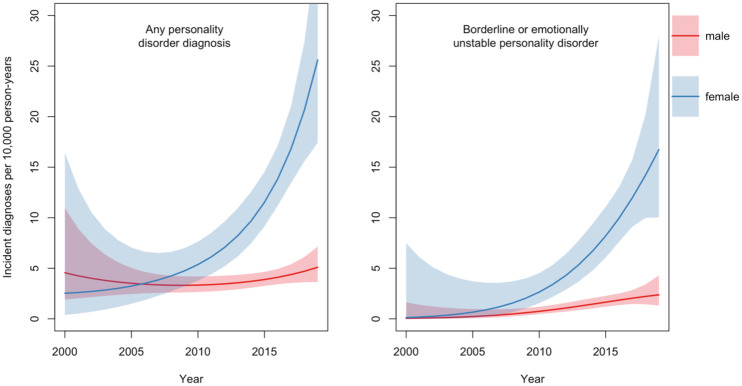
Time trends in the incidence of personality disorder diagnoses, standardised to age 20, from 2000 to 2019, with 95% confidence intervals stratified by sex (males vs females).

We also estimated standardised incidence rates by calendar year with age fixed at 20 for men and women separately, pooling data from Autistic and non-Autistic people (Figure 2). The rate of new personality disorder diagnoses among men stayed broadly similar at 4.6 (95% CI: 1.9–10.9) per 10,000 person-years in 2000 compared to 5.1 (3.6–7.1) in 2019 (Supplemental eTable 2). In women, the rate increased from 2.5 (0.4–16.4) per 10,000 person-years in 2000 to 25.6 (17.4–37.6) in 2019 (Supplemental eTable 2). Rates of new EUPD or BPD diagnoses increased in both groups, with higher rates across the time-period found in women.

## Discussion

We aimed to determine whether diagnosed Autistic adults were more likely to receive a diagnosis of personality disorder compared to other adults of the same age and sex using routinely collected data from UK primary care. For Autistic men and women without ID, the rate of new personality disorder diagnoses was more than four times higher than in comparison adults. For Autistic men with ID, the rate was around twice as high, while for Autistic women with ID, it was >8 times as high. This indicates that even after an autism diagnosis, Autistic people are more likely than the general population to be diagnosed with a personality disorder. Autistic people were also disproportionately likely to have a pre-existing personality disorder diagnosis before their entry into the study.

Women were more likely to receive a new personality disorder or EUPD or BPD diagnosis compared to men, whether Autistic or not (Table 3). The incidence of personality disorder diagnosis increased in women between 2000 and 2019 but remained largely stable in men. For EUPD or BPD diagnoses, there was an increase in both sexes (Figure 2). Pooling data for men and women, the estimated time trend for any personality disorder diagnosis increased in the comparison group between 2000 and 2019. Among Autistic people, the time trend followed a ‘U’ shape function (although the confidence interval is wide in the earlier part of the time-period due to small numbers). This could reflect the intersection of changes in diagnostic trends for both autism and personality disorder. Trends in the comparison group suggest increasing use of personality disorder as a diagnostic term in general, leading to an increase in personality disorder diagnoses in later years in parallel with growing rates of autism diagnoses in people without ID (O’Nions, Petersen, et al., 2023).

Previous work comparing rates of diagnosed autism prevalence to estimated community prevalence suggested that most Autistic adults in England may be undiagnosed (O’Nions, Petersen, et al., 2023). Increasing rates of personality disorder diagnoses in the comparison group could reflect an increasing trend towards undiagnosed Autistic people (represented in the comparison group) receiving this diagnosis in place of an autism diagnosis, particularly women.

The difference in rates of diagnosed personality disorder at cohort entry (i.e. pre-existing diagnoses) relative to the comparison group was higher than the difference in the rate of new personality disorder diagnoses between Autistic and comparison participants. Given that groups were age-matched at cohort entry, this demonstrates that Autistic participants were disproportionately likely to get a personality disorder diagnosis at a younger age than participants in the comparison group. Case reports suggest that Autistic teenage girls are often diagnosed with an ‘emerging’ BPD when they require an inpatient mental health service admission because of self-injurious behaviour, suicidality or eating disorders (Eaton, 2023), conditions that have become more common at a population-level across the time-period studied (Cybulski et al., 2021). The context of these symptoms being attributed to personality disorder is a culture where Autistic people (especially girls and women) need to adapt their behaviour to ‘fit in’, to prevent ridicule and shame, and therefore mask or camouflage their autism, making it harder to identify (Eaton, 2023).

Though not the focus of this study, the approximately 10-fold increase in the rate of new personality disorder diagnoses in Autistic and comparison women combined (but not Autistic and comparison men combined) between 2000 and 2019 depicted in Figure 2 is striking and concerning. Taken together with evidence that people with a personality disorder receive a poorer standard of care (Eaton, 2023; L. Sheehan et al., 2016; Sulzer, 2015), these findings have clear equity-related implications for women and warrant further attention from practitioners, service funders and policy makers.

### Comparison with other studies

A previous meta-analysis indicated a pooled prevalence of 4% for personality disorder diagnoses among Autistic people (Au-Yeung et al., 2019). Data from Denmark using an unmatched cohort design comprising 20,500 autistic people and 1.9 million comparison people born between 1980 and 2010 reported that 3.4% of Autistic people had a personality disorder diagnosis, compared to 1.2% of the comparison group (Schendel et al., 2016). Data from a matched US cohort of 4685 Autistic and 46,850 matched comparison adults aged 65+ reported a rate of 3.1% in Autistic versus 0.1% in comparison adults, a 24-fold difference in odds (Hand, Coury, et al., 2020). However, one study comparing rates of personality disorder diagnosis in nearly 1800 Medicaid-enrolled Autistic people versus 5300 matched comparison adults between 2000 and 2008 reported a prevalence of 2.5% among Autistic people versus 2% in the comparison group.

We found lower rates of pre-existing diagnoses than studies in clinic-based populations accessing mental health support (T. Brugha et al., 2020; G. Rydén et al., 2008), potentially due to differences in sample identification/ascertainment, plus differences in diagnostic practices between countries. The young age at cohort entry for our participants meant there was less opportunity for a personality disorder diagnosis to have been made, since these diagnoses are usually made in adulthood.

### Relevance for policy and research

The present findings highlight a need for efforts to reduce misdiagnosis of autism as personality disorder, with important implications for equity. This could include raising awareness of stress and trauma disproportionately experienced by Autistic people due to adverse experiences (Griffiths et al., 2019), social exclusion (Botha & Frost, 2020) and the cognitive demands associated with masking/camouflaging (Evans et al., 2024), which lead to emotional dysregulation that might be attributed to personality disorder.

Diagnosis of co-occurring mental health conditions (e.g. depression, psychosis, post-traumatic stress disorder) rather than attribution of associated symptoms to personality disorder may improve Autistic people’s access to evidence-based treatment. Formulation of an Autistic person’s emotional support needs as a response to environmental pressures may help the individual understand themselves and be more self-accepting (Au-Yeung et al., 2019). While it has been argued that a personality disorder diagnosis could be useful for personalisation of support for some Autistic individuals (Zavlis & Tyrer, 2024), potential benefits should be weighed against significant costs relating to the increased likelihood of exclusion and stigma.

### Limitations

This study has several limitations. First, the generalisability of our findings may be limited because of bias affecting the sample. Only a small proportion of Autistic adults living in the United Kingdom have been diagnosed. It is likely that undiagnosed people differ from diagnosed populations in important ways. On the one hand, undiagnosed Autistic people may have different support needs (e.g. fewer communication challenges) and are more likely to live independently in the community (T. Brugha et al., 2008) compared to diagnosed Autistic people (e.g. Howlin & Moss, 2012). On the other hand, undiagnosed autism could increase the chances that emotional or self-regulation difficulties are attributed to personality disorder. Population-based studies using survey methodology are needed to estimate rates of personality disorder diagnoses in both diagnosed and undiagnosed Autistic people.

Second, we were unable to examine rates of personality disorder diagnoses in gender-diverse Autistic people, as gender diversity was not coded in the database. This is potentially important given the high rate of personality disorder diagnoses among Autistic trans and gender-diverse young people (Strauss et al., 2021).

Third, our sample included a limited number of participants in earlier years due to the relative rarity of autism diagnoses, and the characteristics of participants are likely to have changed during our period of study. This trend may continue, meaning that our results may not generalise to future cohorts of Autistic people. The relatively small number of diagnosed Autistic participants with an ID, particularly women, meant that the confidence intervals on our estimates were large. A fourth limitation is that the type of personality disorder diagnosis was not available for all personality disorder diagnoses, which together with sample size constraints meant that it was not possible to explore the types of personality disorder diagnoses received and whether these differed between Autistic and comparison participants.

A fifth limitation is the nature of the code-lists used to identify autism and ID. Historical terms such as ‘Heller’s syndrome’ that captured subtypes of pervasive developmental disorder/childhood disintegrative disorder or types of (specific) learning disability from older versions of ICD were included. The rationale was to ensure that older Autistic people were not disproportionately excluded from the sample, and that older diagnoses indicating an ID were not overlooked. These now-obsolete terms may not be recognisable to primary care providers, and some may not precisely match modern diagnostic definitions (e.g. ‘Developmental disorder of scholastic skill’ may have been used to indicate a specific learning difficulty, rather than ID).

Finally, due to the common and non-random missingness of ethnicity information in primary care (Pham et al., 2017), this analysis could not consider whether there were differences in rates of personality disorder diagnoses between people of different ethnicities.

## Conclusion and implications

Autistic people, particularly women, are more likely than the general population to be diagnosed with a personality disorder. The use of personality disorder diagnosis is increasing generally, including in people without a previous autism diagnosis. Autistic people may have support needs that could be misinterpreted as a personality disorder, and there is a need for greater understanding of autism (and particularly of masking in Autistic women and girls) (Gould & Ashton-Smith, 2011; Halladay et al., 2015; Lockwood Estrin et al., 2021) in adult services to prevent misidentification of autism as personality disorder. This has the potential to reduce inequities and lead to more positive outcomes for Autistic people.

## Supplemental Material

sj-docx-1-aut-10.1177_13623613251414911 – Supplemental material for Personality disorder diagnoses in UK Autistic people: Evidence from a matched cohort study Supplemental material, sj-docx-1-aut-10.1177_13623613251414911 for Personality disorder diagnoses in UK Autistic people: Evidence from a matched cohort study by Elizabeth O’Nions, Jude Brown, Joshua EJ Buckman, Rebecca Charlton, Claudia Cooper, Céline El Baou, Francesca Happé, Sarah Hoare, Dan Lewer, Cathie Long, Jill Manthorpe, Douglas GJ McKechnie, Marcus Richards, Rob Saunders, Will Mandy and Joshua Stott in Autism

sj-docx-2-aut-10.1177_13623613251414911 – Supplemental material for Personality disorder diagnoses in UK Autistic people: Evidence from a matched cohort study Supplemental material, sj-docx-2-aut-10.1177_13623613251414911 for Personality disorder diagnoses in UK Autistic people: Evidence from a matched cohort study by Elizabeth O’Nions, Jude Brown, Joshua EJ Buckman, Rebecca Charlton, Claudia Cooper, Céline El Baou, Francesca Happé, Sarah Hoare, Dan Lewer, Cathie Long, Jill Manthorpe, Douglas GJ McKechnie, Marcus Richards, Rob Saunders, Will Mandy and Joshua Stott in Autism

## Notes

1. The term ‘pre-existing diagnosis’ is used to mean diagnosis prior to the beginning of the observation period, rather than (necessarily) prior to the person’s autism diagnosis.

2. In total, 9,504,311 adults had no autism diagnosis recorded at any time prior to or during the observation period (see Supplemental eFigure 1). Across the time-period studied, diagnosed autism prevalence was c. 0.23%.

3. We were also unable to explore rates of personality disorder diagnoses experienced by gender-diverse Autistic people, as gender diversity was not coded in the database.
