# Supplementary material for: Personality disorder diagnoses in UK Autistic people: Evidence from a matched cohort study
Source: Autism. 2026 Feb 27;30(4):901–13. doi: 10.1177/13623613251414911 (PMC13005888; doi:10.1177/13623613251414911)
Supplement: sj-docx-1-aut-10.1177_13623613251414911 – Supplemental material for Personality disorder diagnoses in UK Autistic people: Evidence from a matched cohort study [file sj-docx-1-aut-10.1177_13623613251414911.docx]

**Supplementary Figures for “Personality disorder diagnoses in UK Autistic people: evidence from a matched cohort study”**

Contents

[eFigure 1: Flow diagram for practice/ participant exclusions 2](#_Toc157607645)

[eFigure 2: Simplified schematic description of steps to identify the matched groups & start dates 3](#_Toc157607646)

## eFigure 1: Flow diagram for practice/ participant exclusions

| 794 practices |  | 2 excluded: Missing Acceptable Computer Usage/ Acceptable Mortality Recording date |
| --- | --- | --- |
|  |  |  |
| 792 practices  18,241,856 people |  | 2,517,068 excluded due to poor record quality or not being permanently registered. |
|  |  |  |
| 15,724,788 people |  | 6,163,338 excluded due to no data after the point that the practice met data quality thresholds for electronic recording of patient data, no person-time after 01/01/2000, and/or no person-time after 18^th^ birthday. |
|  |  |  |
| 9,561,450 people |  | 34,821 excluded due to having diagnosed ID prior to cohort entry and no subsequent autism diagnosis (n = 34,648), or a record of autism with no date (n = 173) |
|  |  |  |
| 22,303 people with an autism diagnosis prior to, or during follow-up |  | 9,504,311 people with no autism record at any time; 15 people with a record of autism after the end of follow-up. |
|  |  |  |
| **Autististic people with and without ID**  17,249 people contribute person-time post an autism diagnosis (of whom 1,432 got a subsequent ID diagnosis prior to the end of follow-up. These individuals were excluded from the autism/ID- group, leaving 15,817).  6,486 people contribute person-time post an autism and an ID diagnosis. |  | **Sampling pool for identification of matched participants**  9,504,311 people with no autism record at any time.  3,913 people contribute person-time prior to an autism or an ID diagnosis. |
|  |  |  |
| 22,303 to be matched  191 had insufficient matches |  |  |
|  |  |  |
| **22,112 matched 10:1** |  | **221,120 matches** |

## eFigure 2: Simplified schematic description of steps to identify the matched groups & start dates

The schematics below depict how the matched groups were identified. Rows represent different individuals within a primary care practice, registered and contributing data from 2001 up until 2011 or the point at which they left the practice.

**Step 1:** We allocated person-time (after patient registration and once data quality thresholds have been met) to one of 4 categories, designated by the different colours:

|  | **2001** | **2002** | **2003** | **2004** | **2005** | **2006** | **2007** | **2008** | **2009** | **2010** |  |  | **Key** |  |  |
| --- | --- | --- | --- | --- | --- | --- | --- | --- | --- | --- | --- | --- | --- | --- | --- |
| **A** | **.** |  |  |  |  | **X** |  |  |  |  |  | **.** | **Date of registration** | | |
| **B** | **.** | **X** |  |  |  | **!** |  |  |  |  |  | **X** | **Autism diagnosis** | | |
| **C** | **.** |  |  |  |  |  |  | **X** |  |  |  | **!** | **ID diagnosis** | | |
| **D** | **.** |  |  |  |  |  |  |  |  |  |  |  | **Autism no ID** | | |
| **E** | **.** |  |  | **!** |  |  |  | **X** |  |  |  |  | **Autism with ID** | | |
| **F** | **.** |  |  |  |  |  |  |  |  |  |  |  | ***Potentially* eligible person-time: matched comparison group** | | |
| **G** | **.** |  |  |  |  |  |  | **X!** |  |  |  |  | ***Ineligible person-time*** | | |
| **H** | **.** |  |  |  |  |  |  |  |  |  |  |  |  | | |

*B was diagnosed with ID after being diagnosed Autistic, so could contribute person-time to both the autism/ID- and to the autism/ID+ groups. However, we decided to exclude the person-time prior to B’s ID diagnosis to avoid biasing the estimates for the autism/ID- group.*

**Step 2:** For each Autistic person who had follow-up time without an ID record (A and C), we identified a set of matched people who were in the database & had neither an autism nor an ID record on the date of the Autistic person’s diagnosis. We gave them the same start (“index”) date as their Autistic counterpart (the date of the Autistic person’s autism diagnosis). Those who went on to get an autism or an ID diagnosis were censored from the comparison group prior to their diagnosis.

|  | **2001** | **2002** | **2003** | **2004** | **2005** | **2006** | **2007** | **2008** | **2009** | **2010** |  | |  |  |  |  |
| --- | --- | --- | --- | --- | --- | --- | --- | --- | --- | --- | --- | --- | --- | --- | --- | --- |
| **A** | **.** |  |  |  |  | **X** |  |  |  |  |  | |  |  |  |  |
| **P** | **.** |  |  |  |  |  |  |  |  |  |  | |  |  |  |  |
| **Z** | **.** |  |  |  |  |  |  |  |  |  |  | |  |  |  |  |
| **C** | **.** |  |  |  |  |  |  | **X** |  |  |  |  |  |  |  |  |
| **X** | **.** |  |  |  |  |  |  |  |  |  |  |  |  |  |  |  |
| **P** | **.** |  |  |  |  |  |  |  |  |  |  | |  |  |  |  |

**Step 3:** For each Autistic person who had follow-up time *with* a concurrent ID record (B, E, and G), we sampled a new set of matches by identifying people who were in the database & had neither an autism nor an ID record on the date of the Autistic person’s diagnosis. We gave them the same start (“index”) date as their Autistic counterpart (the latest of the date of their autism or ID diagnosis).

|  | **2001** | **2002** | **2003** | **2004** | **2005** | **2006** | **2007** | **2008** | **2009** | **2010** |  |
| --- | --- | --- | --- | --- | --- | --- | --- | --- | --- | --- | --- |
| **B** | **.** | **X** |  |  |  | **!** |  |  |  |  |  |
| **U** | **.** |  |  |  |  |  |  |  |  |  |  |
| **M** | **.** |  |  |  |  |  |  |  |  |  |  |
| **E** | **.** |  |  | **!** |  |  |  | **X** |  |  |  |
| **S** | **.** |  |  |  |  |  |  |  |  |  |  |
| **X** | **.** |  |  |  |  |  |  |  |  |  |  |
| **G** | **.** |  |  |  |  |  |  | **X!** |  |  |  |
| **L** | **.** |  |  |  |  |  |  |  |  |  |  |
| **K** | **.** |  |  |  |  |  |  |  |  |  |  |
