# Supplementary material for: Personality disorder diagnoses in UK Autistic people: Evidence from a matched cohort study
Source: Autism. 2026 Feb 27;30(4):901–13. doi: 10.1177/13623613251414911 (PMC13005888; doi:10.1177/13623613251414911)
Supplement: sj-docx-2-aut-10.1177_13623613251414911 – Supplemental material for Personality disorder diagnoses in UK Autistic people: Evidence from a matched cohort study [file sj-docx-2-aut-10.1177_13623613251414911.docx]

**eTables: Personality disorder diagnoses in UK Autistic people: evidence from a matched cohort study**

Contents

[eTable 1: Pre-existing conditions at baseline in Autistic people with and without ID and their respective comparison groups. 3](#_Toc176186411)

[eTable 2: Modelled incidence of personality disorder diagnosis in Autistic and non-Autistic people (males and females combined), standardised to age 20. 4](#_Toc176186412)

[eTable 3: Modelled incidence of personality disorder diagnosis in males and females (Autistic and comparison participants combined), standardised to age 20. 7](#_Toc176186413)

# eTable 1: Pre-existing conditions at baseline in Autistic people with and without ID and their respective comparison groups.

|  | Men | | | Women | | |
| --- | --- | --- | --- | --- | --- | --- |
|  | Autistic  n (%) | Comparison  n (%) | Risk ratio (95% CI) | Autistic  n (%) | Comparison  n (%) | Risk ratio (95% CI) |
| ***Autistic people without ID*** | | | | | | |
| Anxiety | 2153 (17.88) | 5670 (4.71) | 3.80 (3.63 - 3.98) | 1117 (30.74) | 3906 (10.75) | 2.86 (2.7 - 3.03) |
| Depression | 2383 (19.79) | 6706 (5.57) | 3.55 (3.4 - 3.71) | 1261 (34.70) | 5277 (14.52) | 2.39 (2.27 - 2.51) |
| Self-harm | 861 (7.15) | 2317 (1.92) | 3.72 (3.44 - 4.01) | 670 (18.44) | 1733 (4.77) | 3.87 (3.56 - 4.20) |
| Harmful alcohol use | 345 (2.87) | 2276 (1.89) | 1.52 (1.36 - 1.69) | 91 (2.50) | 675 (1.86) | 1.35 (1.09 - 1.67) |
| Substance use | 359 (2.98) | 1784 (1.48) | 2.01 (1.8 - 2.25) | 86 (2.37) | 317 (0.87) | 2.71 (2.14 - 3.43) |
| Any personality disorder | 326 (2.71) | 234 (0.19) | 13.93 (11.79 - 16.46) | 221 (6.08) | 148 (0.41) | 14.93 (12.16 - 18.34) |
| Borderline/ emotionally unstable PD | 60 (0.50) | 41 (0.03) | 14.63 (9.84 - 21.76) | 102 (2.81) | 79 (0.22) | 12.91 (9.64 - 17.29) |
| ***Autistic people with ID*** | | | | | | |
| Anxiety | 609 (12.58) | 2643 (5.46) | 2.30 (2.12 - 2.50) | 250 (15.65) | 1839 (11.52) | 1.36 (1.2 - 1.54) |
| Depression | 558 (11.53) | 3595 (7.43) | 1.55 (1.43 - 1.69) | 269 (16.84) | 2714 (16.99) | 0.99 (0.88 - 1.11) |
| Self-harm | 265 (5.48) | 1143 (2.36) | 2.32 (2.04 - 2.64) | 159 (9.96) | 836 (5.23) | 1.9 (1.62 - 2.24) |
| Harmful alcohol use | 83 (1.71) | 1158 (2.39) | 0.72 (0.57 - 0.89) | 21 (1.31) | 286 (1.79) | 0.73 (0.47 - 1.14) |
| Substance use | 61 (1.26) | 833 (1.72) | 0.73 (0.57 - 0.95) | 16 (1.00) | 181 (1.13) | 0.88 (0.53 - 1.47) |
| Any personality disorder | 120 (2.48) | 163 (0.34) | 7.36 (5.83 - 9.30) | 50 (3.13) | 77 (0.48) | 6.49 (4.57 - 9.24) |
| Borderline/ emotionally unstable PD | 20 (0.41) | 25 (0.05) | 8.00 (4.45 - 14.39) | 12 (0.75) | 23 (0.14) | 5.22 (2.6 - 10.47) |

.

# eTable 2: Modelled incidence of personality disorder diagnosis in Autistic and non-Autistic people (males and females combined), standardised to age 20.

| Group (Autistic/ comparison) | Personality disorder type | Year | Modelled rate per 10,000 person-years |
| --- | --- | --- | --- |
| Autistic | any | 2000 | 52.91 (20.42-137.11) |
| Autistic | any | 2001 | 42.45 (19.01-94.75) |
| Autistic | any | 2002 | 34.72 (17.68-68.17) |
| Autistic | any | 2003 | 28.96 (16.40-51.12) |
| Autistic | any | 2004 | 24.63 (15.16-40.00) |
| Autistic | any | 2005 | 21.35 (13.96-32.67) |
| Autistic | any | 2006 | 18.88 (12.82-27.80) |
| Autistic | any | 2007 | 17.02 (11.81-24.53) |
| Autistic | any | 2008 | 15.65 (10.98-22.31) |
| Autistic | any | 2009 | 14.67 (10.36-20.77) |
| Autistic | any | 2010 | 14.02 (9.98-19.71) |
| Autistic | any | 2011 | 13.67 (9.83-19.00) |
| Autistic | any | 2012 | 13.58 (9.93-18.58) |
| Autistic | any | 2013 | 13.76 (10.27-18.45) |
| Autistic | any | 2014 | 14.22 (10.82-18.68) |
| Autistic | any | 2015 | 14.98 (11.54-19.45) |
| Autistic | any | 2016 | 16.10 (12.29-21.07) |
| Autistic | any | 2017 | 17.63 (12.95-24.00) |
| Autistic | any | 2018 | 19.69 (13.46-28.81) |
| Autistic | any | 2019 | 22.43 (13.86-36.30) |
| Autistic | bpd | 2000 | 1.26 (0.04-38.68) |
| Autistic | bpd | 2001 | 1.46 (0.08-27.33) |
| Autistic | bpd | 2002 | 1.69 (0.14-20.12) |
| Autistic | bpd | 2003 | 1.95 (0.25-15.45) |
| Autistic | bpd | 2004 | 2.25 (0.41-12.38) |
| Autistic | bpd | 2005 | 2.59 (0.65-10.37) |
| Autistic | bpd | 2006 | 2.97 (0.97-9.11) |
| Autistic | bpd | 2007 | 3.41 (1.38-8.40) |
| Autistic | bpd | 2008 | 3.90 (1.86-8.15) |
| Autistic | bpd | 2009 | 4.45 (2.38-8.32) |
| Autistic | bpd | 2010 | 5.06 (2.90-8.84) |
| Autistic | bpd | 2011 | 5.76 (3.43-9.66) |
| Autistic | bpd | 2012 | 6.53 (4.01-10.64) |
| Autistic | bpd | 2013 | 7.39 (4.68-11.68) |
| Autistic | bpd | 2014 | 8.35 (5.49-12.71) |
| Autistic | bpd | 2015 | 9.42 (6.44-13.79) |
| Autistic | bpd | 2016 | 10.60 (7.38-15.25) |
| Autistic | bpd | 2017 | 11.91 (7.96-17.82) |
| Autistic | bpd | 2018 | 13.35 (7.92-22.51) |
| Autistic | bpd | 2019 | 14.94 (7.33-30.47) |
| comparison | any | 2000 | 0.99 (0.27-3.60) |
| comparison | any | 2001 | 1.08 (0.36-3.27) |
| comparison | any | 2002 | 1.19 (0.47-3.03) |
| comparison | any | 2003 | 1.31 (0.60-2.86) |
| comparison | any | 2004 | 1.45 (0.76-2.75) |
| comparison | any | 2005 | 1.60 (0.95-2.70) |
| comparison | any | 2006 | 1.77 (1.15-2.70) |
| comparison | any | 2007 | 1.96 (1.38-2.77) |
| comparison | any | 2008 | 2.17 (1.62-2.90) |
| comparison | any | 2009 | 2.41 (1.87-3.11) |
| comparison | any | 2010 | 2.68 (2.13-3.38) |
| comparison | any | 2011 | 2.98 (2.40-3.71) |
| comparison | any | 2012 | 3.33 (2.70-4.10) |
| comparison | any | 2013 | 3.72 (3.06-4.52) |
| comparison | any | 2014 | 4.16 (3.47-4.98) |
| comparison | any | 2015 | 4.66 (3.95-5.50) |
| comparison | any | 2016 | 5.22 (4.45-6.13) |
| comparison | any | 2017 | 5.87 (4.91-7.01) |
| comparison | any | 2018 | 6.60 (5.26-8.28) |
| comparison | any | 2019 | 7.44 (5.49-10.07) |
| comparison | bpd | 2000 | 0.01 (0.00-0.72) |
| comparison | bpd | 2001 | 0.02 (0.00-0.68) |
| comparison | bpd | 2002 | 0.04 (0.00-0.65) |
| comparison | bpd | 2003 | 0.06 (0.00-0.64) |
| comparison | bpd | 2004 | 0.08 (0.01-0.65) |
| comparison | bpd | 2005 | 0.13 (0.02-0.67) |
| comparison | bpd | 2006 | 0.18 (0.05-0.70) |
| comparison | bpd | 2007 | 0.26 (0.09-0.76) |
| comparison | bpd | 2008 | 0.37 (0.16-0.84) |
| comparison | bpd | 2009 | 0.51 (0.27-0.95) |
| comparison | bpd | 2010 | 0.69 (0.43-1.12) |
| comparison | bpd | 2011 | 0.92 (0.62-1.35) |
| comparison | bpd | 2012 | 1.19 (0.85-1.67) |
| comparison | bpd | 2013 | 1.52 (1.11-2.08) |
| comparison | bpd | 2014 | 1.89 (1.41-2.54) |
| comparison | bpd | 2015 | 2.32 (1.78-3.03) |
| comparison | bpd | 2016 | 2.79 (2.19-3.56) |
| comparison | bpd | 2017 | 3.28 (2.55-4.22) |
| comparison | bpd | 2018 | 3.79 (2.74-5.26) |
| comparison | bpd | 2019 | 4.29 (2.68-6.88) |

Note: any = any personality disorder diagnosis. Bpd = EUPD or BPD diagnosis

# eTable 3: Modelled incidence of personality disorder diagnosis in males and females (Autistic and comparison participants combined), standardised to age 20.

| Sex: male (m) or female (f) | Personality disorder type | Year | Modelled rate per 10,000 person-years |
| --- | --- | --- | --- |
| m | any | 2000 | 4.55 (1.90-10.94) |
| m | any | 2001 | 4.25 (2.03-8.93) |
| m | any | 2002 | 4.01 (2.15-7.45) |
| m | any | 2003 | 3.80 (2.27-6.37) |
| m | any | 2004 | 3.64 (2.37-5.59) |
| m | any | 2005 | 3.51 (2.46-5.03) |
| m | any | 2006 | 3.42 (2.52-4.64) |
| m | any | 2007 | 3.36 (2.56-4.40) |
| m | any | 2008 | 3.32 (2.59-4.26) |
| m | any | 2009 | 3.31 (2.62-4.20) |
| m | any | 2010 | 3.33 (2.65-4.19) |
| m | any | 2011 | 3.38 (2.71-4.22) |
| m | any | 2012 | 3.46 (2.80-4.27) |
| m | any | 2013 | 3.57 (2.92-4.35) |
| m | any | 2014 | 3.71 (3.08-4.46) |
| m | any | 2015 | 3.89 (3.26-4.64) |
| m | any | 2016 | 4.11 (3.42-4.93) |
| m | any | 2017 | 4.38 (3.55-5.40) |
| m | any | 2018 | 4.71 (3.62-6.12) |
| m | any | 2019 | 5.10 (3.64-7.14) |
| m | bpd | 2000 | 0.05 (0.00-1.63) |
| m | bpd | 2001 | 0.07 (0.00-1.40) |
| m | bpd | 2002 | 0.09 (0.01-1.22) |
| m | bpd | 2003 | 0.13 (0.01-1.10) |
| m | bpd | 2004 | 0.17 (0.03-1.02) |
| m | bpd | 2005 | 0.23 (0.05-0.97) |
| m | bpd | 2006 | 0.30 (0.09-0.94) |
| m | bpd | 2007 | 0.38 (0.15-0.95) |
| m | bpd | 2008 | 0.48 (0.24-0.99) |
| m | bpd | 2009 | 0.60 (0.34-1.07) |
| m | bpd | 2010 | 0.74 (0.46-1.19) |
| m | bpd | 2011 | 0.90 (0.59-1.37) |
| m | bpd | 2012 | 1.07 (0.72-1.58) |
| m | bpd | 2013 | 1.25 (0.87-1.82) |
| m | bpd | 2014 | 1.45 (1.03-2.05) |
| m | bpd | 2015 | 1.65 (1.20-2.27) |
| m | bpd | 2016 | 1.85 (1.37-2.50) |
| m | bpd | 2017 | 2.04 (1.46-2.84) |
| m | bpd | 2018 | 2.22 (1.44-3.40) |
| m | bpd | 2019 | 2.37 (1.31-4.29) |
| f | any | 2000 | 2.53 (0.39-16.42) |
| f | any | 2001 | 2.60 (0.52-12.93) |
| f | any | 2002 | 2.71 (0.70-10.53) |
| f | any | 2003 | 2.84 (0.91-8.89) |
| f | any | 2004 | 3.02 (1.17-7.77) |
| f | any | 2005 | 3.24 (1.49-7.05) |
| f | any | 2006 | 3.51 (1.85-6.64) |
| f | any | 2007 | 3.84 (2.27-6.50) |
| f | any | 2008 | 4.25 (2.73-6.62) |
| f | any | 2009 | 4.75 (3.23-7.00) |
| f | any | 2010 | 5.37 (3.78-7.63) |
| f | any | 2011 | 6.12 (4.41-8.50) |
| f | any | 2012 | 7.06 (5.19-9.61) |
| f | any | 2013 | 8.23 (6.18-10.96) |
| f | any | 2014 | 9.69 (7.47-12.56) |
| f | any | 2015 | 11.53 (9.15-14.53) |
| f | any | 2016 | 13.85 (11.20-17.13) |
| f | any | 2017 | 16.83 (13.45-21.05) |
| f | any | 2018 | 20.65 (15.57-27.38) |
| f | any | 2019 | 25.60 (17.43-37.60) |
| f | bpd | 2000 | 0.13 (0.00-7.47) |
| f | bpd | 2001 | 0.18 (0.01-6.08) |
| f | bpd | 2002 | 0.25 (0.01-5.11) |
| f | bpd | 2003 | 0.35 (0.03-4.44) |
| f | bpd | 2004 | 0.48 (0.06-3.99) |
| f | bpd | 2005 | 0.66 (0.12-3.71) |
| f | bpd | 2006 | 0.89 (0.22-3.57) |
| f | bpd | 2007 | 1.19 (0.39-3.56) |
| f | bpd | 2008 | 1.57 (0.66-3.70) |
| f | bpd | 2009 | 2.05 (1.05-4.00) |
| f | bpd | 2010 | 2.65 (1.55-4.52) |
| f | bpd | 2011 | 3.39 (2.16-5.31) |
| f | bpd | 2012 | 4.29 (2.88-6.41) |
| f | bpd | 2013 | 5.38 (3.72-7.78) |
| f | bpd | 2014 | 6.67 (4.76-9.36) |
| f | bpd | 2015 | 8.19 (6.06-11.07) |
| f | bpd | 2016 | 9.95 (7.61-13.00) |
| f | bpd | 2017 | 11.96 (9.11-15.69) |
| f | bpd | 2018 | 14.22 (9.99-20.24) |
| f | bpd | 2019 | 16.73 (10.02-27.93) |

Note: any = any personality disorder diagnosis. Bpd = EUPD or BPD diagnosis
